# Drug Resistance Mechanisms in *Mycobacterium tuberculosis*

**DOI:** 10.3390/antibiotics3030317

**Published:** 2014-07-02

**Authors:** Juan Carlos Palomino, Anandi Martin

**Affiliations:** Laboratory of Microbiology, Department of Biochemistry and Microbiology, Ghent University, K.L. Ledeganckstraat 35, 9000 Gent, Belgium; E-Mail: Anandi.Martin@ugent.be

**Keywords:** drug resistance, molecular mechanisms, *Mycobacterium tuberculosis*

## Abstract

Tuberculosis (TB) is a serious public health problem worldwide. Its situation is worsened by the presence of multidrug resistant (MDR) strains of *Mycobacterium tuberculosis*, the causative agent of the disease. In recent years, even more serious forms of drug resistance have been reported. A better knowledge of the mechanisms of drug resistance of *M. tuberculosis* and the relevant molecular mechanisms involved will improve the available techniques for rapid drug resistance detection and will help to explore new targets for drug activity and development. This review article discusses the mechanisms of action of anti-tuberculosis drugs and the molecular basis of drug resistance in *M. tuberculosis*.

## 1. Introduction

Tuberculosis (TB) remains as an important infectious disease and public health concern worldwide. According to the latest World Health Organization (WHO) report, there were an estimated 8.6 million incident cases of TB in 2012 and 1.3 million deaths were attributed to the disease. More than half a million cases occurred in children and 320,000 deaths were reported among HIV-infected persons [1]. However, even more disturbing is the emergence of drug resistance. In 2012, there were an estimated 450,000 cases of multidrug resistant (MDR)-TB and 170,000 deaths were due to it. MDR-TB is caused by strains of *Mycobacterium tuberculosis* that are resistant to at least rifampicin and isoniazid, two key drugs in the treatment of the disease. Since 2006, it has been recognized the presence of even more resistant strains of *M. tuberculosis* labelled as extensively drug resistant (XDR)-TB [2,3,4]. These strains in addition to being MDR are also resistant to any fluoroquinolone and to at least one of the injectable second-line drugs: kanamycin, capreomycin or amikacin. More recently, a more worrying situation has emerged with the description of *M. tuberculosis* strains that have been found resistant to all antibiotics that were available for testing, a situation labelled as totally drug resistant (TDR)-TB [5,6,7]. Early detection of all forms of drug resistance in TB is a key factor to reduce and contain the spread of these resistant strains. A better knowledge of the mechanisms of action of anti-TB drugs and the development of drug resistance will allow identifying new drug targets and better ways to detect drug resistance. The following sections will review the mode of action and resistance mechanisms of the main anti-TB drugs as well as new drugs recently described with anti-TB activity.

## 2. First-Line Anti-TB Drugs

### 2.1. Rifampicin

Rifampicin is a rifamycin derivative introduced in 1972 as an antituberculosis agent. It is one of the most effective anti-TB antibiotics and together with isoniazid constitutes the basis of the multidrug treatment regimen for TB. Rifampicin is active against growing and non-growing (slow metabolizing) bacilli [8]. The mode of action of rifampicin in *M. tuberculosis* is by binding to the β-subunit of the RNA polymerase, inhibiting the elongation of messenger RNA [9]. The majority of rifampicin-resistant clinical isolates of *M. tuberculosis* harbor mutations in the *rpoB* gene that codes for the β-subunit of the RNA polymerase. As a result of this, conformational changes occur that decrease the affinity for the drug and results in the development of resistance [10].

In about 96% of *M. tuberculosis* isolates resistant to rifampicin, there are mutations in the so-called “hot-spot region” of 81-bp spanning codons 507–533 of the *rpoB* gene. This region is also known as the rifampicin resistance-determining region [11]. Mutations in codons 516, 526 and 531 are the most commonly associated mutations with rifampicin resistance in the majority of studies [12,13]. Although less frequent, some reports have also noted the occurrence of mutations outside of the hot-spot region of *rpoB* [14,15]. Cross-resistance with other rifamycins can occur. Mutations in some codons (e.g., 518 or 529) have been associated with low-level resistance to rifampicin but still susceptible to other rifamycins, such as rifabutin or rifalazil [16,17]. This is important for TB patients that need to receive antiretroviral therapy since rifabutin is a less effective inducer of the cytochrome P450 CYP3A oxidative enzyme [18]. On the other hand, monoresistance to rifampicin is quite rare and almost all rifampicin-resistant strains are also resistant to other drugs, especially to isoniazid. This is the reason why rifampicin resistance is considered as a surrogate marker for MDR-TB [19].

Recent genome sequencing studies have uncovered the acquisition of compensatory mutations in *rpoA* and *rpoC*, encoding the α and β' subunits of RNA polymerase, in rifampicin-resistant strains with mutations in *rpoB* [20]. These compensatory mutations would be responsible for restoring the fitness of these strains *in vivo* and have also been associated with a higher transmissibility in some settings [21,22].

### 2.2. Isoniazid

Isoniazid was introduced in 1952 as an anti-TB agent and it remains, together with rifampicin, as the basis for the treatment of the disease. Unlike rifampicin, isoniazid is only active against metabolically-active replicating bacilli. Also known as isonicotinic acid hydrazide, isoniazid is a pro-drug that requires activation by the catalase/peroxidase enzyme KatG, encoded by the *katG* gene, to exert its effect [23]. Isoniazid acts by inhibiting the synthesis of mycolic acids through the NADH-dependent enoyl-acyl carrier protein (ACP)-reductase, encoded by *inhA* [24]. Although simple in its structure, resistance to this drug has been associated with mutations in several genes, such as *katG*, *inhA*, *ahpC*, *kasA* and NDH.

The two main molecular mechanisms of isoniazid resistance are associated with gene mutations in *katG* and *inhA* or its promoter region. Indeed, numerous studies have found mutations in these two genes as the most commonly associated with isoniazid resistance [25,26]. Among these, the most prevalent gene mutation has been identified as S315T in *katG* resulting in an isoniazid product deficient in forming the isoniazid-NAD adduct needed to exert its antimicrobial activity [27,28]. This mutation has been consistently associated with high-level resistance (MIC > 1 µg/mL) to isoniazid [29] and occurs more frequently in MDR strains [26]. The second most common mutation occurs in the promoter region of *inhA* causing an overexpression of InhA or less frequently, a mutation in its active site, which decreases its affinity for the isoniazid-NAD adduct [28]. The most prevalent mutation found is at position −15C/T and is more commonly associated with low level resistance to isoniazid (MIC < 1 µg/mL). Mutations in *inhA* not only cause resistance to isoniazid but also to the structurally related drug ethionamide, which shares the same target [30,31]. A recent study found that a mutation in the *inhA* regulatory region together with a mutation in the *inhA* coding region produced high-level isoniazid resistance and also cross-resistance to ethionamide [32].

One recent interesting finding showed that the 4R isomer of the isoniazid-NADP adduct causes inhibition of the dihydrofolate reductase (DfrA) in *M. tuberculosis*, suggesting that mutations in *dfrA* could possibly play a role in resistance to isoniazid [33]. Moreover, an analysis of the proteome of isoniazid targets in *M. tuberculosis* identified 16 other proteins, in addition to InhA and DfrA, that were bound by these adducts with high affinity, which could signal other not yet clearly defined actions of isoniazid on the bacteria [34]. Two recent studies, however, have failed to identify any mutation in *dfrA* associated with resistance to isoniazid [35,36].

In *M. tuberculosis*, *ahpC* encodes an alkyl hydroperoxidase reductase that is implicated in resistance to reactive oxygen intermediates and it was initially proposed that mutations in the promoter of *ahpC* could be used as proxy markers for isoniazid resistance [37]. It is now better understood that mutations in the promoter of *ahpC* are compensatory mutations for the loss of catalase/peroxidase activity rather than the cause for isoniazid resistance [38]. Moreover, overexpression of AhpC does not confer resistance to isoniazid [39].

Several studies have found single nucleotide polymorphisms in other genes in isoniazid resistant clinical isolates of *M. tuberculosis*, including *kasA* and the *oxyR*-*ahpC* and *furA*-*katG* intergenic regions [26,40,41]. However, their direct role as a cause of isoniazid resistance has not been fully demonstrated. On the other hand, co-resistance to isoniazid and ethionamide has been clearly demonstrated to be caused by mutations in ndh in M. smegmatis and M. bovis BCG, by altering the NADH/NAD ratios inside the cell, leading to a competitive inhibition of the INH-NAD adduct [42,43]. A recent study has also found that a silent mutation in mabA conferred isoniazid resistance through upregulation of *inhA* in *M. tuberculosis* [44].

### 2.3. Ethambutol

Ethambutol was first introduced in the treatment of TB in 1966 and is part of the current first-line regimen to treat the disease. Ethambutol is bacteriostatic against multiplying bacilli interfering with the biosynthesis of arabinogalactan in the cell wall [45]. In *M. tuberculosis*, the genes *embCAB*, organized as an operon, code for arabinosyl transferase, which is involved in the synthesis of arabinogalactan, producing the accumulation of the intermediate d-arabinofuranosyl-P-decaprenol [46].

The recognized mechanism of resistance to ethambutol has been linked to mutations in the gene *embB* with mutations at position *embB*306 as the most prevalent in most of the studies performed [47,48]. Some studies, however, have also found mutations in *embB*306 in ethambutol susceptible isolates [49]. Moreover, a study with a large number of *M. tuberculosis* isolates found that mutations in *embB*306 were not necessarily associated with resistance to ethambutol but with a predisposition to develop resistance to increasing number of drugs and to be transmitted [50]. In fact, allelic exchange studies have shown that individual mutations causing certain amino acid substitutions produced ethambutol resistance, while other amino acid substitutions had little or no effect on ethambutol resistance [51]. The same authors have more recently reported that mutations in the decaprenylphosphoryl-B-d-arabinose (DPA) biosynthetic and utilization pathway genes, Rv3806c and Rv3792, together with mutations in *embB* and *embC* accumulate, giving rise to a range of MICs of ethambutol depending on mutation type and number [52]. These findings could have influence on the correct detection of ethambutol resistance by current molecular methods. Mutations in *embB*306 then, cause variable degrees of ethambutol resistance and are required but are not enough to cause high-level resistance to ethambutol. There remain about 30% ethambutol resistant strains that do not present any mutation in embB stressing the need to identify other possible mechanisms of drug resistance to this drug.

### 2.4. Pyrazinamide

Pyrazinamide was introduced into TB treatment in the early 1950s and constitutes now part of the standard first-line regimen to treat the disease. Pyrazinamide is an analog of nicotinamide and its introduction allowed reducing the length of treatment to six months. It has the characteristic of inhibiting semi-dormant bacilli residing in acidic environments such as found in the TB lesions [53]. Pyrazinamide is also a pro-drug that needs to be converted to its active form, pyrazinoic acid, by the enzyme pyrazinamidase/nicotinamidase coded by the *pncA* gene [54,55]. The proposed mechanism of action of pyrazinamide involves conversion of pyrazinamide to pyrazinoic acid, which disrupts the bacterial membrane energetics inhibiting membrane transport. Pyrazinamide would enter the bacterial cell by passive diffusion and after conversion to pyrazinoic acid it is excreted by a weak efflux pump. Under acid conditions, the protonated pyrazinoic acid would be reabsorbed into the cell and accumulated inside, due to an inefficient efflux pump, resulting in cellular damage [56]. One study has also found that pyrazinoic acid and its n-propyl ester can inhibit the fatty acid synthase type I in replicating *M. tuberculosis* bacilli [57,58].

A recent study, however, has challenged the previous model by proposing that pyrazinoic acid inhibits trans-translation, a process of ribosome-sparing in *M. tuberculosis* [59]. The study was performed in pyrazinamide-resistant strains lacking mutations in pncA but that had mutations in *rpsA* identifying the ribosomal protein 1 (RpsA) as the proposed target. Overexpression of RpsA conferred increased resistance to pyrazinamide and pyrazinoic acid was confirmed to be bound to RpsA [59]. While a very intriguing hypothesis as a target for pyrazinamide, the failure to perform allelic transfers in this study makes it difficult to conclude that in fact mutations in *rpsA* are the target of pyrazinamide.

Mutations in the gene *pncA* remain as the most common finding in pyrazinamide resistant strains. These mutations, however, are scattered throughout the gene but most occur in a 561-bp region in the open reading frame or in an 82-bp region of its putative promoter [60,61]. Some few studies have reported the occurrence of pyrazinamide resistant strains without any mutation in pncA stating that the resistance could be due to mutations in another not yet identified regulatory gene [62]. Based on the current evidence, the contribution of mutations in *rpsA* to pyrazinamide resistance remains limited [63,64,65].

### 2.5. Streptomycin

Originally isolated from the soil microorganism Streptomyces griseus, streptomycin was the first antibiotic to be successfully used against TB. Unfortunately, as soon as it was prescribed, resistance to it emerged, a result of being administered as monotherapy [66]. Streptomycin is an aminocyclitol glycoside active against actively growing bacilli and its mode of action is by inhibiting the initiation of the translation in the protein synthesis [67]. More specifically, streptomycin acts at the level of the 30S subunit of the ribosome at the ribosomal protein S12 and the 16S rRNA coded by the genes *rpsL* and *rrs*, respectively [68].

Consequently, mutations in *rpsL* and rrs are the major mechanisms of resistance to streptomycin but account for 60%–70% of the resistance found [69]. Among the mutations reported in *rpsL*, a substitution in codon 43 from lysine to arginine has been the most commonly reported. This mutation produces high-level resistance to streptomycin. In *rrs* the most common mutations occur around nucleotides 530 and 915. There remain an important percentage of strains resistant to streptomycin that lack mutations in either of these two genes, suggesting additional mechanisms of resistance.

In the last years, it has also been reported that mutations in *gidB*, a gene encoding a conserved 7-methylguanosine methyltransferase specific for the 16S rRNA, confers low-level resistance to streptomycin [70,71].

## 3. Second-Line Anti-TB Drugs

### 3.1. Fluoroquinolones

Fluoroquinolones are currently in use as second-line drugs in the treatment of MDR-TB. Both ciprofloxacin and ofloxacin are synthetic derivatives of the parent compound nalidixic acid, discovered as a by-product of the antimalarial chloroquine [72]. Newer-generation quinolones such as moxifloxacin and gatifloxacin are being evaluated in clinical trials and proposed as first-line antibiotics with the purpose of shortening the length of treatment in TB [73,74].

The mode of action of fluoroquinolones is by inhibiting the topoisomerase II (DNA gyrase) and topoisomerase IV, two critical enzymes for bacterial viability. These proteins are encoded by the genes *gyrA*, *gyrB*, *parC* and *parE*, respectively [75]. In *M. tuberculosis*, only type II topoisomerase (DNA gyrase) is present and, thus, is the only target of fluoroquinolone activity [76]. Type II topoisomerase is a tetramer formed by two α and β subunits, coded by *gyrA* and *gyrB*, respectively, which catalyzes the supercoiling of DNA [77]. The main mechanism of development of fluoroquinolone resistance in *M. tuberculosis* is by chromosomal mutations in the quinolone resistance-determining region of *gyrA* or *gyrB*. The most frequent mutations found are at position 90 and 94 of *gyrA* but mutations at position 74, 88 and 91 have also been reported [78,79]. A recent systematic review of fluoroquinolone-resistance-associated gyrase mutations in *M. tuberculosis* has been published [80].

One interesting finding in *M. tuberculosis* is the presence of a natural polymorphism at position 95 in *gyrA* that is not related to fluoroquinolone resistance since it is also found in fluoroquinolone-susceptible strains [81]. Another interesting finding has been the report that the simultaneous occurrence of mutations T80A and A90G in *gyrA* led to hypersusceptibility to several quinolones [82]. This finding could point out that the problem of fluoroquinolone resistance in *M. tuberculosis* might be more complex than was thought initially.

Cross-resistance is assumed to occur between fluoroquinolones although isolated reports have acknowledged the presence of strains resistant to gatifloxacin and moxifloxacin that were still susceptible to ofloxacin [83]. Also, the involvement of efflux mechanisms has been suggested as a possible cause for fluoroquinolone resistance in *M. tuberculosis* [84].

### 3.2. Kanamycin, Capreomycin, Amikacin, Viomycin

These four antibiotics have the same mechanism of action by inhibiting the protein synthesis but, while kanamycin and amikacin are aminoglycosides, capreomycin and viomycin are cyclic peptide antibiotics. All four are second-line drugs used in the management of MDR-TB.

Kanamycin and amikacin inhibit protein synthesis by alteration at the level of 16S rRNA. The most common mutations found in kanamycin-resistant strains are at position 1400 and 1401 of the *rrs* gene, conferring high-level resistance to kanamycin and amikacin. However, mutations at position 1483 have also been reported [85,86]. Full cross-resistance between kanamycin and amikacin is not complete, as previously thought. Some studies have shown variable levels and patterns of resistance suggesting that other mechanisms of resistance might be possible [87]. In concordance with this, a low-level resistance to kanamycin has been associated with mutations in the promoter region of the *eis* gene, encoding an aminoglycoside acetyltransferase [88]. Mutations at position −10 and −35 of the *eis* promoter led to an overexpression of the protein and low-level resistance to kanamycin but not to amikacin. These mutations were found in up to 80% of clinical isolates showing low-level resistance to kanamycin [88,89].

Capreomycin and viomycin, on the other hand, have a similar structure and bind at the same site in the ribosome, at the interface of the small and large subunits [90]. They show full cross-resistance as reported in previous studies [91]. Mutations in the *tlyA* gene have also been associated with resistance to capreomycin and viomycin. TlyA is an rRNA methyltransferase specific for 2'-O-methylation of ribose in rRNA. Mutations in *tlyA* determine the absence of methylation activity [92]. Although some studies did not find this association, a recent meta-analysis, evaluating the association of genetic mutations and resistance to second-line drugs, has confirmed the presence of *tlyA* mutations in addition to mutations in *rrs* and *eis* [93].

### 3.3. Ethionamide

Ethionamide is a derivative of isonicotinic acid structurally similar to isoniazid. It is also a pro-drug requiring activation by a monooxygenase encoded by the ethA gene. It interferes with the mycolic acid synthesis by forming an adduct with NAD that inhibits the enoyl-ACP reductase enzyme. EthA is regulated by the transcriptional repressor EthR [94]. Resistance to ethionamide occurs by mutations in *etaA*/*ethA*, *ethR* and also mutations in *inhA*, which cause resistance to both isoniazid and ethionamide [95,96]. Moreover, studies with spontaneous isoniazid- and ethionamide-resistant mutants of *M. tuberculosis* found that they map to mshA, encoding an enzyme essential for mycothiol biosynthesis [97].

### 3.4. Para-Amino Salicylic Acid

Although it was one of the first anti-tuberculosis drugs used in the treatment of the disease, together with isoniazid and streptomycin, para-amino salicylic acid or PAS is now considered as a second-line drug part of the treatment regimen for MDR-TB. Until recently, its mechanism of action was not completely defined. It has been proposed that being an analog of para-amino benzoic acid, it must compete with it for dihydropteroate synthase, interfering in the process of folate synthesis. A study using transposon mutagenesis identified mutations in the thyA gene associated with resistance to PAS that were also present in clinical isolates resistant to PAS [98]. A recent study has also identified various missense mutations in folC encoding dihydrofolate synthase that conferred resistance to PAS in laboratory isolates of *M. tuberculosis* [99]. In a panel of 85 clinical MDR-TB isolates, mutations in *folC* were identified in five isolates resistant to PAS. Nevertheless, just less than 40% of PAS-resistant strains had mutations in thyA indicating that still other mechanisms of resistance to the drug might exist [100].

### 3.5. Cycloserine

Cycloserine is an oral bacteriostatic second-line anti-tuberculosis drug used in MDR-TB treatment regimens. It is an analog of d-alanine that by blocking the activity of d-alanine: d-alanine ligase inhibits the synthesis of peptidoglycan. It can also inhibit d-alanine racemase (AlrA) needed for the conversion of l-alanine to d-alanine [101]. Although the actual target of cycloserine in *M. tuberculosis* is not completely elucidated, in previous studies in *M. smegmatis* it was shown that overexpression of *alrA* led to resistance to cylcoserine in recombinant mutants [102]. More recently, it has also been shown that a point mutation in cycA, which encodes a d-alanine transporter, was partially responsible for resistance to cycloserine in *M. bovis* BCG [103].

### 3.6. Thioacetazone

Thioacetazone is an old drug that was used in the treatment of TB due to its favourable *in vitro* activity against *M. tuberculosis* and its very low cost. It has toxicity problems, however, especially in patients co-infected with HIV. It belongs to the group 5 drugs of the WHO and acts by inhibiting mycolic acid synthesis [104].

### 3.7. Macrolides

Macrolides are more frequently recommended for the treatment of other mycobacterial infections due to their limited activity against *M. tuberculosis*. Among them, clarithromycin is considered as part of the group 5 drugs of the WHO. Intrinsic resistance to macrolides has been attributed to low cell wall permeability and the expression of *emr37*, a gene that codifies for a methylase at a specific site in the 23S rRNA, blocking the binding of the antibiotic. In studies performed with *M. tuberculosis* and Mycobacterium microti it was found that this intrinsic resistance was inducible with sub-inhibitory concentrations of clarithromycin, leading to four- to eight-fold increase in MIC values [105]. Moreover, in studies performed with clinical isolates of *M. tuberculosis*, sub-inhibitory concentrations of ethambutol reversed resistance to clarithromycin, signalling a permeability barrier as the cause of the intrinsic resistance to the macrolide [106].

### 3.8. Clofazimine

Clofazimine is a riminophenazine compound reported long ago as having anti-TB activity [107]. Due to the availability of other effective anti-TB drugs at the time and some undesirable side-effects, such as pigmentation of the skin, its use was more limited to the treatment of leprosy [108]. It is now considered in the group 5 drugs of the WHO for the management of MDR-TB. Until recently, the exact mode of action of this antibiotic was not completely understood. Recent studies, however, have signalled the outer membrane as the possible target of clofazimine [109]. Another study found that in *M. tuberculosis* clofazimine is reduced by NADH dehydrogenase and subsequently after spontaneous reoxidation liberates bactericidal levels of reactive oxygen species (ROS) [110].

Resistance to clofazimine has not yet been fully characterized; however, a recent study has found that in spontaneous mutants of the reference strain H37Rv, mutations in the transcriptional regulator Rv0678 caused an upregulation of MmpL5, a multisubstrate efflux pump, which not only caused resistance to clofazimine but also to bedaquiline [111].

### 3.9. Linezolid

Also part of the category 5 drugs of second-line anti-TB drugs, linezolid is an oxazolidinone originally approved for clinical use in the treatment of skin infections and nosocomial pneumonia caused by Gram-positive bacteria [112]. The mode of action of linezolid is by inhibition of an early step in the synthesis of proteins, binding to the 50S ribosomal subunit [101]. Resistance to linezolid in *M. tuberculosis* is still unusual, but a study analyzing 210 MDR strains found 1.9% of strains being resistant to linezolid [113]. Further analysis of *in vitro* selected linezolid-resistant mutants found that strains with mutations in the 23S rRNA had MICs of 16–32 µg/mL, while strains with MICs of 4–8 µg/mL or susceptible strains showed no mutations [114]. A more recent study using next-generation sequencing has also found the mutation T460C in *rplC*, encoding the 50S ribosomal L3 protein, in *in vitro*-selected mutants and clinical isolates of *M. tuberculosis* resistant to linezolid [115]. Previous studies have also found evidence of the possible involvement of efflux pumps in the resistance of *M. tuberculosis* to linezolid [84].

Table 1 gives an overview of the first- and second-line anti-tuberculosis drugs currently in use and target of action.

**Table 1:** First- and second-line TB drugs, genes involved in their activation and mechanisms involved.

Drug	Gene	Mechanism Involved	Reference
Isoniazid	*katG*, *inhA*	Catalase/peroxidase; enoyl reductase	[26]
Rifampicin	*rpoB*	RNA polymerase	[11,12,13]
Pyrazinamide	*pncA*, *rpsA*	Pyrazinamidase; ribosomal protein 1	
Ethambutol	*embB*	Arabinosyl transferase	[40,41]
Streptomycin	*rpsL*, *rrs*, *gidB*	S12 ribosomal protein, 16A rRNA, 7-methylguanosine methyltransferase	[59,60,61]
Quinolones	*gyrA*, *gyrB*	DNA gyrase	[67,68]
Capreomycin	*rrs*, *tlyA*	16S rRNA, rRNA methyltransferase	[80,81]
Kanamycin/Amikacin	*rrs*	16S rRNA	[83]
Ethionamide	*ethA*	Enoyl-ACP reductase	[85,86]
Para-aminosalicylic acid	*thyA*, *folC*	Thymidylate synthase A	[87,88,89]

## 4. New Anti-TB Drugs

Notwithstanding the alleged lack of interest of the pharmaceutical industry for the development of new antibiotics, there are several anti-tuberculosis drugs in the pipeline and some of them are already being evaluated in clinical trials and in new combinations with the purpose of reducing the length of TB treatment.

### 4.1. Bedaquiline

Formerly known as TMC207 or R207910, bedaquiline is a new antibiotic belonging to the class of diarylquinolines with specific activity against *M. tuberculosis*, which has also shown *in vitro* activity against other non-tuberculous mycobacteria [116]. Bedaquiline was discovered after a high-throughput evaluation of thousands of compounds using *Mycobacterium smegmatis* in a whole-cell assay [117]. The drug showed *in vitro* and *in vivo* activity against *M. tuberculosis* and then entered into clinical evaluation for drug susceptible and MDR-TB [74,118,119]. Based on the results of two phase II clinical trials, bedaquiline has recently received conditional approval for the treatment of MDR-TB under the trade name Sirturo. A “black box” warning is, however, accompanying this authorization due to the reported unexplained deaths and QT interval prolongation. Recent reviews and evaluation of this new drug have been published [120,121]. A phase III clinical trial was scheduled to begin in 2013 but has not yet started. Bedaquiline is also being evaluated in new combination regimens with the purpose of reducing the length of treatment [122].

**Figure 1:**
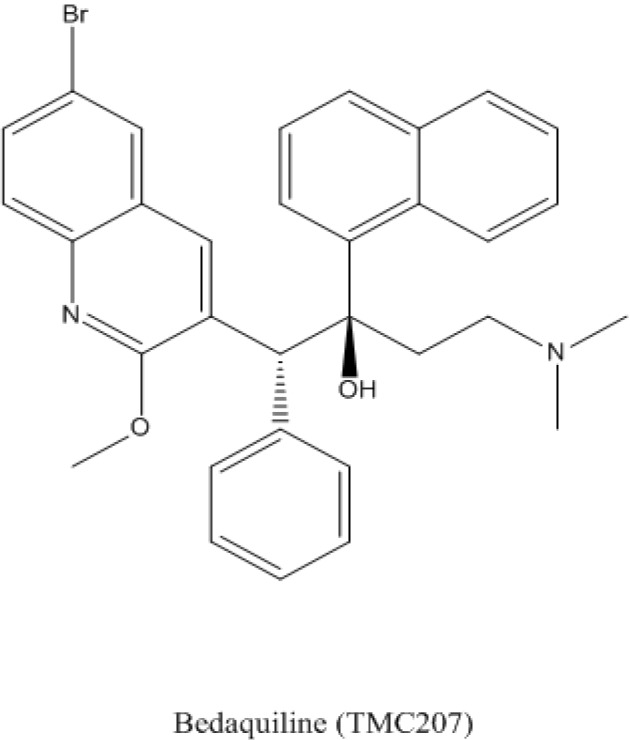
Structure of bedaquiline.

The mode of action of bedaquiline is by inhibiting the ATP synthase of *M. tuberculosis*, which was a completely new target of action for an antimycobacterial drug. This mode of action was discovered by analyzing *M. tuberculosis* and *M. smegmatis* mutants resistant to bedaquiline. By sequencing the genome of these mutants and comparing to that of the susceptible strains, the only mutation found was in the *atpE* gene, which encodes the c part of the F0 subunit of the ATP synthase [117]. This is a complex structure that generates the ATP needed by the mycobacterial cell [123] for which bedaquiline has a favored specificity compared to mitochondrial ATP synthase [124]. The structure of bedaquiline is shown in Figure 1.

The most prevalent mutation in the *atpE* gene found in bedaquiline resistant mutants is A63P but also I66M has been found. The latter introduces a modification that interferes the proper binding of bedaquiline to its target [125,126]. Nevertheless, in a study to further assess the mechanisms of resistance to bedaquiline in *M. tuberculosis*, it was found that only 15 out of 53 resistant mutants had mutations in *atpE*. The other 38 strains lacked mutations in *atpE* or even in the F0 or F1 operons, which suggests that other mechanisms of resistance are still possible [127].

### 4.2. Delamanid

**Figure 2:**
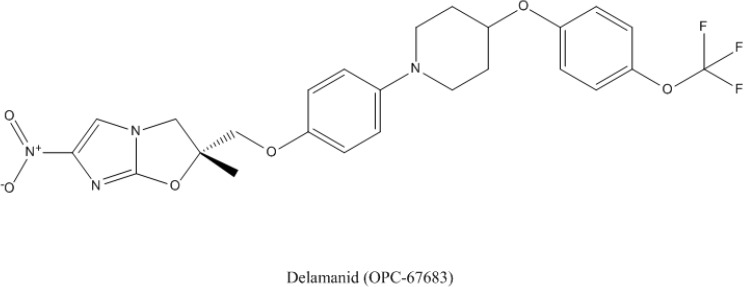
Structure of delamanid.

Delamanid, previously known as OPC-67683, is a derivative of nitro-dihydro-imidazooxazole with activity against *M. tuberculosis* that acts by inhibiting the synthesis of mycolic acid and is undergoing clinical evaluation in a phase III trial [74]. The structure of delamanid is shown in Figure 2. Delamanid was previously shown to have a very good *in vitro* and *in vivo* activity against drug-susceptible and drug-resistant *M. tuberculosis* [128], as well as good early bactericidal activity comparable to that of rifampicin [129]. Delamanid has more recently shown its safety and efficacy in a clinical evaluation for MDR-TB [130].

The specific mode of action of delamanid is by inhibition of the mycolic acid synthesis but it differs from isoniazid in that, it only inhibits methoxy- and keto-mycolic acid while isoniazid also inhibits α-mycolic acid [128].

Delamanid also requires reductive activation by *M. tuberculosis* to exert its activity. In experimentally generated delamanid-resistant mycobacteria, a mutation was found in the Rv3547 gene, suggesting its role in the activation of the drug [128].

### 4.3. PA-824

**Figure 3:**
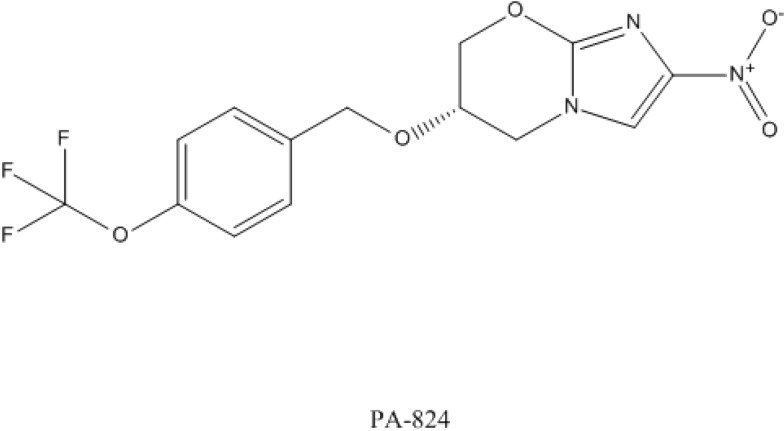
Structure of PA-824.

PA-824 is a bicyclic derivative of nitroimidazole that showed specific activity against *M. tuberculosis* [131]. The structure of PA-824 is shown in Figure 3. This small-molecule compound showed a very good *in vitro* and *in vivo* activity in animal models [132] and it also showed to be safe and well tolerated [133]. PA-824 is currently undergoing further clinical evaluations [122,134].

PA-824 needs to be activated by a nitroreductase to exert its activity and it inhibits the synthesis of protein and cell wall lipids [131]. The mechanism of resistance to PA-824 has been shown to be most commonly associated with loss of a specific glucose-6-phosphate dehydrogenase (FGD1) or the dezaflavin cofactor F420. More recently, a nitroimidazo-oxazine-specific protein causing minor structural changes in the drug has also been identified [135].

### 4.4. SQ-109

**Figure 4:**
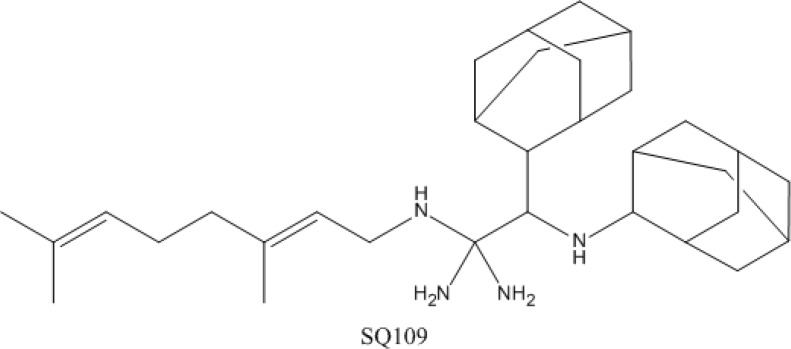
Structure of SQ-109.

Compound SQ-109 is a synthetic analogue of ethambutol that has shown *in vitro* and *in vivo* activity against drug-susceptible and drug-resistant *M. tuberculosis* [136]. The structure of SQ-109 is shown in Figure 4. It has also been shown to possess synergistic *in vitro* activity when combined with first-line drugs, and more interestingly, when combined with bedaquiline and the oxazolidinone PNU-10048 [137,138,139]. SQ-109 is currently being evaluated in a phase II clinical trial [74].

The mode of action of SQ-109 is by interfering with the assembly of mycolic acids into the bacterial cell wall core, resulting in accumulation of trehalose monomycolate, a precursor of the trehalose dimycolate. Transcriptional studies have shown that, similar to other cell wall inhibitors such as isoniazid and ethambutol, SQ-109 induces the transcription of the iniBAC operon required for efflux pump functioning [140]. Moreover, by producing spontaneously generated resistant mutants to SQ-109 analogs and performing whole-genome sequencing, mutations in the *mmpL3* gene were identified, suggesting MmpL3 as the target of SQ-109 and signaling MmpL3 as transporter of trehalose monomycolate [141].

### 4.5. Benzothiazinones

**Figure 5:**
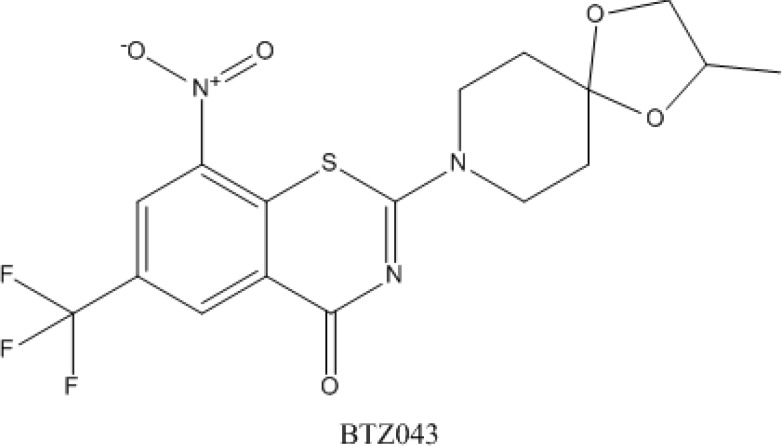
Structure of BTZ043.

A new class of drug with antimycobacterial activity, 1,3-benzothiazin-4-one or benzothiazinone (BTZ), was recently described [142]. The lead compound, 2-[2-S-methyl-1,4-dioxa-8-azaspiro[4.5]dec-8-yl]-8-nitro-6-(trifluoromethyl)-4H-1,3-benzothiazin-4-one (BTZ043) was found to have *in vitro*, *ex vivo* and *in vivo* activity against *M. tuberculosis*. It was also found to be active against drug-susceptible and MDR clinical isolates of *M. tuberculosis* [143]. Structure of BTZ043 is shown in Figure 5.

By transcriptome analysis, the mode of action of BTZ043 was initially spotted at the cell wall biogenesis level. By further genetic analysis, using *in vitro* generated mutants, the target of the drug was identified at the level of the gene rv3790, which together with rv3791 encode proteins that catalyze the epimerization of decaprenylphosphoryl ribose (DPR) to decaprenylphosphoryl arabinose (DPA), a precursor for arabinan synthesis needed for the bacterial cell wall [144]. DprE1 and DprE2 were proposed as names for these two key enzymes [142]. More recent studies have characterized more precisely the mechanism of action of BTZ043 by showing that the drug is activated in the bacteria through reduction of an essential nitro group to a nitroso derivative, which can react with a cysteine residue in DprE1 [145]. In studies with *M. smegmatis*, an alternative mechanism of resistance has been suggested. The overexpression of a nitroreductase NfnB led to the inactivation of the drug by reducing an essential nitro group to an amino group [146]. Although *M. tuberculosis* apparently lacks nitroreductases able to reduce the drug, this finding could be important for development of new BTZ analogues with improved activity.

Just recently a series of piperazine-containing BTZs has been reported. The lead compound PBTZ169 has improved activity, safety and efficacy in animal models and has shown *in vitro* synergy with bedaquiline signaling it as an attractive new candidate for further clinical development [147].

## 5. Concluding Remarks

Drug resistance in TB remains a man-made phenomenon. It emerges as a result of spontaneous gene mutations in *M. tuberculosis* that render the bacteria resistant to the most commonly used anti-TB drugs. Among the reasons for this, the non-compliance with the treatment regimens is signaled as the first cause. The standard treatment of TB calls for a six-month regimen of four drugs that in the case of MDR-TB is extended to 18–24 months involving second-line drugs. This makes compliance with the treatment regimens very challenging and the rates of non-adherence could be high, resulting in poor outcomes and further dissemination of MDR strains.

Notwithstanding the fact that mutations in a number of genes are clearly associated with drug resistance in *M. tuberculosis*, there are still many cases where resistant strains do not harbor any known mutation. For example, a recent study using whole-genome sequencing identified new genes and intergenic regions that were associated with drug resistance and its evolution, showing that TB drug resistance is a phenomenon more complex than previously assumed [148]. More clarification is needed on the role of specific gene mutations and the development of MDR- or XDR-TB, or the relation between drug resistance and fitness of the bacteria. A better knowledge is also required on the role of efflux pump mechanisms and the development of clinical drug resistance, or the role of porins, if any, on the intrinsic resistance to certain antibiotics. It is, thus, quite important to further our knowledge of additional mechanisms of drug resistance to the available anti-TB drugs. This could have a major impact on the dynamics of TB transmission and for the discovery and development of new anti-TB drugs.
